# Brain Amyloid-β Peptide Is Associated with Pain Intensity and Cognitive Dysfunction in Osteoarthritic Patients

**DOI:** 10.3390/ijms252312575

**Published:** 2024-11-22

**Authors:** Chun-Hsien Wen, Hong-Yo Kang, Julie Y.H. Chan

**Affiliations:** 1 Graduate Institute of Clinical Medical Sciences, Chang Gung University, Taoyuan 33302, Taiwan; hkang3@gap.cgu.edu.tw; 2 Department of Anesthesiology, Kaohsiung Veterans General Hospital, Kaohsiung 813414, Taiwan; 3 Department of Nursing, Shu-Zen Junior College of Medicine and Management, Kaohsiung 82144, Taiwan; 4 Department of Nursing, Meiho University, Pingtung 912009, Taiwan; 5 School of Medicine, National Yang Ming Chiao Tung University, Taipei 112304, Taiwan; 6 Division of Endocrinology and Metabolism, Department of Internal Medicine, Kaohsiung Chang Gung Memorial Hospital, Kaohsiung 833401, Taiwan; 7 College of Medicine, Chang Gung University, Taoyuan 33302, Taiwan; 8 Institute for Translational Research in Biomedicine, Kaohsiung Chang Gung Memorial Hospital, Kaohsiung 833401, Taiwan

**Keywords:** amyloid β_40_, amyloid β_42_, cognition, osteoarthritis, pain, pro-inflammatory cytokine/chemokine

## Abstract

Considerable studies have demonstrated that osteoarthritis (OA) is a risk factor for dementia. The precise mechanisms underlying the association between OA and increased risk for cognitive dysfunction, however, remain unclear. This study aimed at exploring the associations between pro-inflammatory cytokines/chemokines, biomarkers of Alzheimer’s disease (AD), pain intensity, and cognitive decline in knee joint OA patients. A total of 50 patients (26 in OA group and 24 in non-OA control group) were enrolled in this prospective, observational study. The visual analogue scale (VAS) score for pain intensity and Cognitive Abilities Screening Instrument (CASI) score for cognitive functions were examined in both groups. The plasma and cerebrospinal fluid (CSF) levels of pro-inflammatory molecules (IL-1β, IL-6, TNF-α, fractalkine, BDNF, MCP-1, and TGF-β), as well as biomarkers of AD (Aβ_40_, Aβ_42_, total-tau, and phospho-tau), were measured by multiplex immunoassay. Correlations among plasma or CSF biomarkers and questionnaire scores were assessed using Pearson’s correlation coefficient and simple linear regressions. There were more patients in the OA group whose CASI cutoff percentiles were <P5 or at P5 than in the control group. VAS pain scores were negatively correlated with cognitive domains, including total score, short term memory, attention, mental manipulation, abstract thinking, and judgment, of the CASI score. VAS scores were positively correlated with fractalkine, Aβ_40_, and Aβ_42_ in CSF of OA patients. The CSF levels of Aβ_40_ and Aβ_42_ in OA patients were negatively correlated with attention and abstract scores in CASI. The findings of this study suggest that knee OA is associated with poor cognitive performance, and this association is particularly pronounced in OA patients with chronic pain. Higher levels of brain AD biomarkers, such as Aβ_40_ and Aβ_42_, may partially mediate this relationship.

## 1. Introduction

Chronic pain is a major health burden that impacts more than 100 million adults and costs over $600 billion dollars annually in the developed countries [1]. Osteoarthritis (OA) pain is one of the most frequent types of chronic pain, and the knee is the most frequently affected joint in up to 10% of men and 13% of women aged above 60 years with symptomatic OA [2,3]. OA is a degenerative joint disease resulting from stresses initiated by any joint or periarticular tissue abnormality [3]. It is characterized by progressive deterioration and loss of articular cartilage with concomitant structural and functional changes in the entire joint, including the synovial membrane, periarticular ligaments, capsule, and subchondral bone [3,4]. The incidence of OA is influenced by many factors, such as work, sports participation, musculoskeletal injuries, overweight or obesity status, gender, older age, history of knee injury, and joint anatomy abnormalities [5,6,7]. The symptoms and main complaints of knee OA include chronic pain, stiffness, reduced joint motion, and muscle weakness [5,6,8,9]. Long-term consequences of knee OA include decreased physical activity, sleep disturbance, fatigue, depression, and disability [3,6,7,10]. With the combined effects of aging and increasing prevalence of obesity in the global population, OA pain has become a health burden with wide socio-economic impact.

Substantial evidence from preclinical and human studies demonstrates that OA is also a risk factor for cognitive deficits [11,12,13]. The prevalence of dementia in patients with OA is higher than that in individuals without OA [14]. Furthermore, neuroimaging studies have revealed alterations in brain areas engaged in cognition. For example, altered hippocampal functional connectivity and decreases in volumes of hippocampal and gray matter of the whole brain over time could be identified in OA patients [15,16,17]. The precise mechanisms underlying the association between OA and the increased risk for cognitive dysfunction, nonetheless, remain elusive. One possible mechanism proposed in previous studies is neuroinflammation attributed by OA-induced increase in the production of pro-inflammatory cytokines and chemokines [11,12,13,15,16,17].

Neuroinflammation is increasingly considered a major cause of neurodegenerative diseases such as Alzheimer’s disease (AD) [18,19,20,21]. The core cerebrospinal fluid (CSF) biomarkers for AD pathophysiology are amyloid-β peptides (Aβ_40_ and Aβ_42_), total-tau (t-tau), and phosphorylated tau (p-tau), which are believed to have the highest diagnostic accuracy for early AD diagnosis [22,23]. Several studies have reported higher levels of neuroinflammatory mediators in blood and CSF of OA patients and their associations with pain, anxiety, depression, and sleep disorders [24,25]. However, none of the previous studies attempted to investigate the CSF biomarkers of AD in OA patients and the associations among the neuroinflammatory mediators, pain intensity, and cognitive functions.

In this study, we aimed to explore the associations among pro-inflammatory molecules, CSF biomarkers of AD, pain intensity, and cognitive functions in knee OA patients. Our results suggest that knee OA is associated with cognitive decline, and this association is particularly pronounced in OA patients with chronic pain. Higher levels of brain AD biomarkers, such as Aβ_40_ and Aβ_42_, may partially mediate this relationship.

## 2. Results

### 2.1. Participants’ Characteristics

Twenty-six knee OA patients at a median age of 68.58 years (range, 58– 86 years) and twenty-four patients in the control group at a median age of 63 years (range, 37–83) were recruited in the study. Demographic characteristics and comorbidities data of the patients are listed in Table 1. There was no significant difference between the two groups regarding age, marital status, body weight, smoking, drinking, or comorbidity diseases. OA patients were constituted of more females, with higher BMI, and received fewer education years than patients in the control group.

### 2.2. Pain and Cognition Assessments

Table 2 illustrates the VAS pain scores in both groups for which the score was significantly higher in the OA group than in the control group (2.38 ± 0.852 vs. 0.21 ± 0.509, *p* < 0.001). In addition, there were more patients in the OA group whose CASI cutoff percentile score was <P5 [7 (26.9%) vs. 1 (4.2%), *p* = 0.028] or at P5 [4 (15.4%) vs. 0 (0%), *p* = 0.045] when compared to the non-OA control group. The cutoff percentile score of lower than P5 is regarded as cognitive function below the normal limit of the same age and education level [24]. There is no apparent difference in VAS or total CASI score between the male and female participants in both OA (VAS: 2.00 ± 0.93 vs. 2.56 ± 0.78, *p* = 0.127; CASI: 89.80 ± 12.12 vs. 79.87 ± 15.26, *p* = 0.118) and non-OA groups (0.18 ± 0.50 vs. 0.50 ± 0.71, *p* = 0.409; CASI: 94.46 ± 5.83 vs. 94.25 ± 2.5, *p* = 0.960).

### 2.3. Biomarker Levels in Plasma and CSF

**Figure 1:**
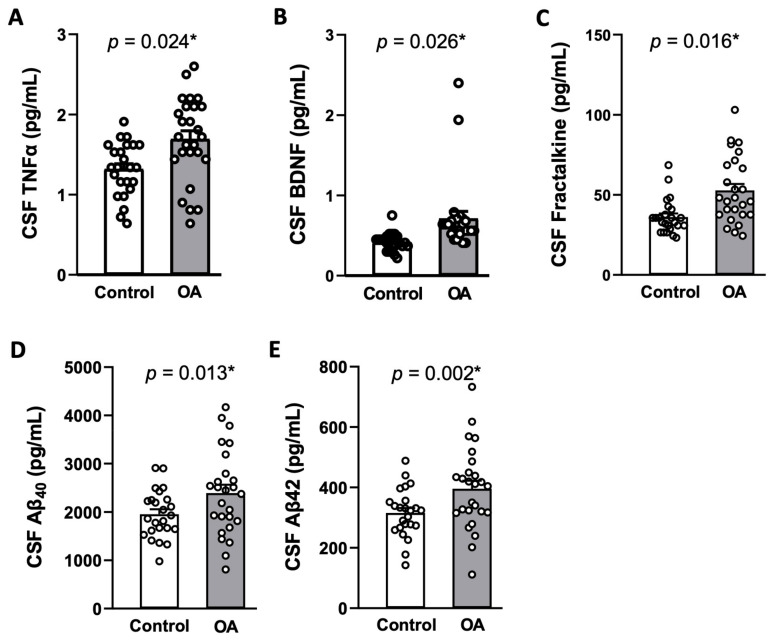
The levels of TNF-α (**A**), BDNF (**B**), fractalkine (**C**), Aβ_40_ (**D**), and Aβ_42_ (**E**) in the cerebral fluid (CSF) of non-OA control patients (n = 24) and OA patients (n = 26). Aβ_40_, amyloid-β_40_; Aβ_42_, amyloid-β_42_; BDNF, brain-derived neurotrophic factor; TNF-α, tumor necrosis factor alpha. Data are means ± SD. * Statistically significant vs. non-OA control group in unpaired Student’s *t*-test after adjustment for covariates with a multivariate analysis of covariance.

As shown in Table 3, most tested cytokines and chemokines in plasma were not discernibly different between the two groups. However, levels of TNF-α, BDNF, fractalkine, Aβ_40_, and Aβ_42_ in CSF were significantly higher in OA patients compared to the control group after adjusting for covariates [Table 4 and Figure 1]. Plasma levels of the tested AD biomarkers were below the detection limit in both groups. Again, no significant difference in plasma or CSF concentrations of cytokines/chemokines was observed between the male and female participants in both OA and non-OA groups [Tables S1 and S2 in Supplementary Materials].

### 2.4. Association Between VAS Pain Score and CASI Cognition Score

**Figure 2:**
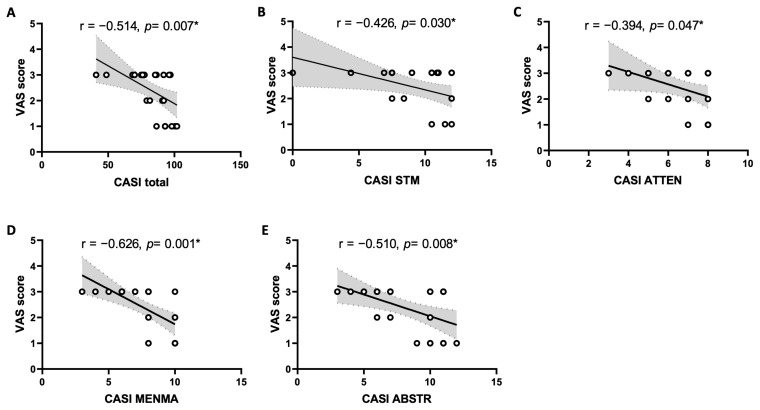
Scatter plot of the visual analogue scale (VAS) pain scores and total score (**A**), as well as short-term memory (STM) (**B**), attention (ATTEN) (**C**), mental manipulation (MENMA) (**D**), and abstract thinking and judgment (ABSTR) (**E**) domains of Cognitive Abilities Screening Instrument (CASI) scores in OA patients (n = 26). The solid lines represent the slope of Pearson correlation coefficient and the dashed lines and shaded region represent the 95% confidence interval. * Statistically significant by Pearson correlation coefficient and simple linear regression analysis.

Pearson’s correlation and simple linear regression analysis were used to analyze the association between the VAS pain score and all domains of the CASI score in OA patients. Table 5 and Figure 2 show that the VAS pain scores were negatively correlated with total score (*r* = −0.514, 95% CI = −0.752, −0.158, *p* = 0.007), short-term memory (*r* = −0.426, 95% CI = −0.698, −0.046, *p* = 0.030), attention (*r* = −0.394, 95% CI = −0.678, −0.008, *p* = 0.047), mental manipulation (*r* = −0.626, 95% CI = −0.816, −0.315, *p* = 0.001), and abstract thinking and judgment (*r* = −0.510, 95% CI = −0.749, −0.153, *p* = 0.008) domains of the CASI score. In contrast, there was no significant correlation between the VAS pain score with the total score or any cognitive domain of CASI scores in the control group [Table S3 in Supplementary Materials].

### 2.5. Association Between VAS Score and Level of CSF Biomarkers

**Figure 3:**
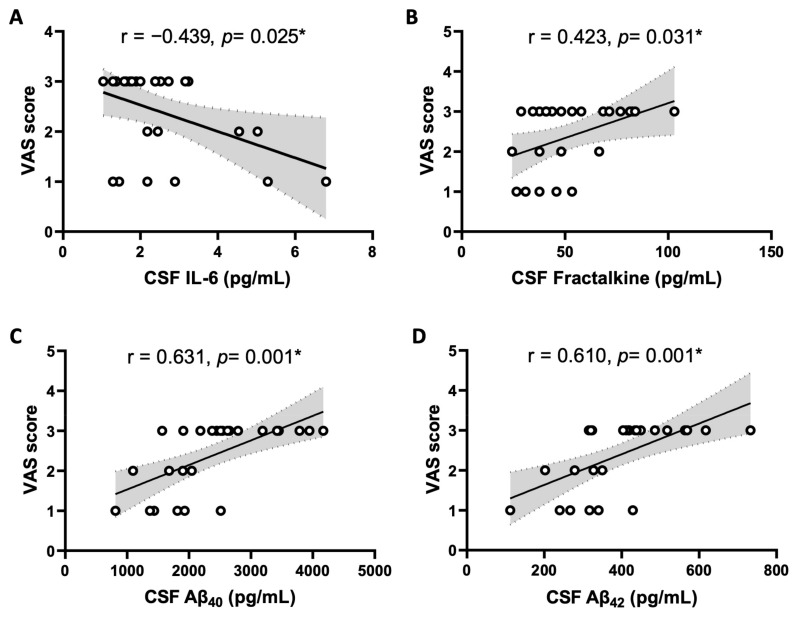
Scatter plot of the visual analogue scale (VAS) pain scores and the cerebral spinal fluid (CSF) concentration of IL-6 (**A**), fractalkine (**B**), Aβ_40_ (**C**), and Aβ_42_ (**D**) in OA patients (n = 26). The solid lines represent the slope of Pearson correlation coefficient and the dashed lines and shaded region represent the 95% confidence interval. * Statistically significant by Pearson correlation coefficient and simple linear regression analysis.

Table 6 and Figure 3 show that the VAS pain scores were negatively correlated with IL-6 (*r* = −0.439, 95% CI = −0.706, −0.062, *p* = 0.025), whereas they were positively correlated with fractalkine (*r* = 0.423, 95% CI = 0.043, 0.696, *p* = 0.031), Aβ_40_ (*r* = 0.631, 95% CI = 0.323, 0.818, *p* = 0.001), Aβ_42_ (*r* = 0.610, 95% CI = 0.292, 0.807, *p* = 0.001) levels in CSF of OA patients. Again, no apparent correlation was observed in the control group, except a positive correlation of CSF TGF-β (*r* = 0.439, 95% CI = 0.044, 0.716, *p* = 0.032) level with VAS score [Table S4 in Supplementary Materials]. In addition, there is no correlation between plasma cytokine/chemokine levels and VAS pain score in control patients [Table S4 in Supplementary Materials].

### 2.6. Association Between CASI Scores and Level of CSF Biomarkers

**Figure 4:**
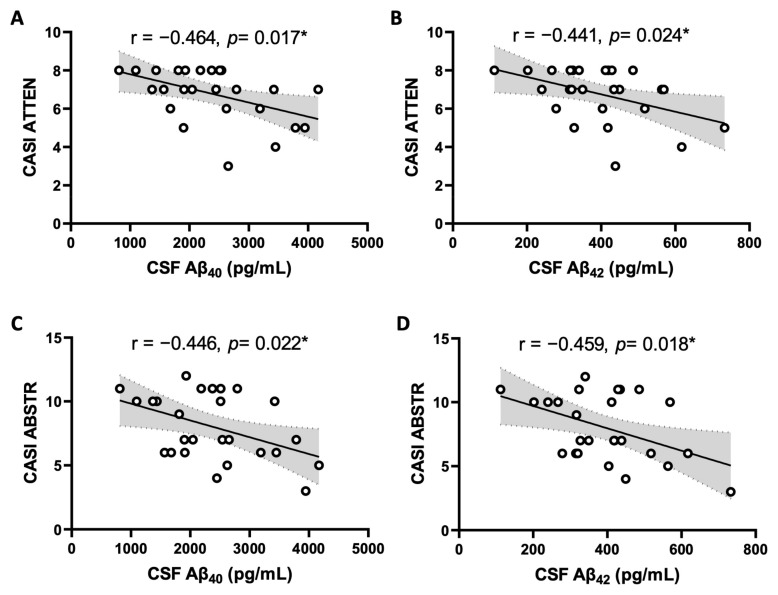
Scatter plot of mental attention (ATTEN) (**A**,**B**) and abstract thinking and judgment (ABSTR) (**C**,**D**) domains of Cognitive Abilities Screening Instrument (CASI) scores and CSF concentration of Aβ_40_ and Aβ_42_ in OA patients (n = 26). The solid lines represent the slope of Pearson correlation coefficient and the dashed lines and shaded region represent the 95% confidence interval. * Statistically significant by Pearson correlation coefficient and simple linear regression analysis.

Table 7 and Figure 4 show that Aβ_40_ in CSF was negatively correlated with attention scores (r = −0.464, *p* = 0.017) and abstract scores (r = −0.446, *p* = 0.022); likewise, levels of CSF Aβ_42_ were negatively correlated with attention scores (r = −0.441, *p* = 0.024) and abstract scores (r = −0.459, *p* = 0.018) of CASI in OA patients. In contrast, no significant correlation between CSF pro-inflammatory cytokine/chemokine levels and CASI score was observed in OA [Table S5 in Supplementary Materials] or control [Table S6 in Supplementary Materials] patients. In addition, CSF AD biomarkers were not correlated with CASI score in control subjects [Table S7 in Supplementary Materials].

## 3. Discussion

To the best of our knowledge, this is the first study to explore the associations among pro-inflammatory molecules, AD biomarkers, pain intensity, and cognitive function in patients of knee OA. The key observations of our study are as follows: (1) a higher proportion of knee OA patients exhibited poor cognitive performance, compared to the non-OA patients after adjustments of educational years and age; (2) VAS pain intensity of OA patients was negatively correlated with cognitive CASI scores (total score, short term memory, attention, mental manipulation, abstract thinking, and judgment); (3) levels of pro-inflammatory cytokine/chemokine (TNF-α/fractalkine) and AD biomarkers (Aβ_40_ and Aβ_42_) in CSF, but not plasma, were higher in OA patients and were positively correlated with pain intensity of the patients; and (4) AD biomarkers, but not pro-inflammatory molecules, in CSF were negatively correlated with attention and abstract domains of CASI scores in OA patients. We interpreted these data to suggest that poor cognitive performance is associated with knee OA, and this association is particularly pronounced in OA patients with chronic pain. Our data also imply that CSF AD biomarkers, such as Aβ_40_ and Aβ_42_, may partially mediate this relationship.

Accumulative evidence from animal and human studies indicates that OA is a risk factor for dementia [11,12,13,15,26], although mechanisms underlying the association have not been fully investigated. As such, one of the major contributions of this study is the identification of a positive correlation between pain intensity with cognitive impairment in OA patients. These findings are consistent with those demonstrated in large scale population-based studies [12,13,27]. Our findings that OA pain impairs the majority of the tested cognitive domains of the CASI, including total score, short-term memory, attention, mental manipulation, and abstract thinking and judgment, suggest a close relationship with neural circuitry for nociception and cognition processing in the central nervous system (CNS). In this regard, the mesocorticolimbic circuitry and the hippocampal formation have been placed as central players for both [28]. Cognition and nociception activate the same brain circuitries in the CNS [29,30]. Chronic pain leads to cognitive dysfunction that is accompanied by structural and functional changes in the hippocampus [31] and disruption of fronto-hippocampal connectivity [32]. Neuroimaging studies further revealed that altered hippocampal functional connectivity and a decrease in volumes of hippocampus over time could be identified in OA patients [15,16,17].

In the present study, we observed higher CSF levels of TNF-α, BDNF, fractalkine, Aβ_40_, and Aβ_42_ in OA participants after covariate adjustments. Among them, the concentrations of fractalkine, Aβ_40_, and Aβ_42_ were positively correlated with the pain intensity in knee OA. Fractalkine, also known as CX_3_CL_1_, is one of the key chemokines that possess pronociceptive properties [33] and increases in a variety of chronic pain conditions, including those associated with OA [34]. Through activation of its receptor CX_3_CR_1_, fractalkine activates neuroimmune crosstalk in the CNS and contributes to the initiation and maintenance of pain in OA [35]. Literature on a positive association of CSF Aβ_40_ and Aβ_42_ with pain, on the other hand, is relatively scarce. As such, another notable contribution of the present study is the evidence of a positive association between the increased CSF Aβ_40_ and Aβ_42_ levels and pain intensity in knee OA. Brain Aβ peptides may cause hyperalgesia by activating microglia [36,37,38], leading to neuroinflammation and the subsequence secretion of nociceptive cytokines and chemokines in the CNS [18]. At a molecular level, production of oxidative biomarkers and activation of intracellular redox-sensitive signals have been postulated to predispose Aβ-treated animals to increase pain sensitivity [39]. In addition to soluble amyloid peptides, misfolded tau, truncated tau, and hyperphosphorylated tau were reported to be accompanied by proliferation of microglia and amplified expression of the inflammatory genes [40], although we did not find significant differences in CSF t-tau and p-tau levels in OA patients. TNF-α is rapidly upregulated in neurons, microglia, and astrocytes following tissue injury and plays a critical role in the generation of central sensitization and persistent pain [41]. At the same time, BDNF is a crucial neuromodulator in pain transmission both in the peripheral nervous system and the CNS [42]. Our observation of a lack of association between CSF levels of TNF-α and BDNF with pain intensity in knee OA is consistent with previous findings [43] and implies that the two factors might not be major contributors to pain intensity in knee OA. Given its small sample size in the present study, the significance of TNF-α and BDNF in chronic pain of OA patients nonetheless warrants further investigation. We noted a negative correlation between CSF IL-6 and VAS pain score, despite its level being higher in OA patients. This discrepancy is not immediately clear and might be related to a small number of OA participants in the study.

Both Aβ_40_ and Aβ_42_ are well-defined biomarkers for AD; their roles in the context of OA-associated cognitive dysfunction, nonetheless, have not been unraveled. Here, we present the first human observation of a positive association of these soluble amyloid peptides with poor cognitive performance of OA patients, particularly in those suffering chronic pain. A higher prevalence of cognitive dysfunction in the OA group than that in the non-OA group has been reported previously [14]. We extend these findings and provide new observations to suggest that higher CSF Aβ_40_ and Aβ_42_ could at least be partly related to cognitive impairment in OA patients. In light of the role of Aβ peptides in pathogenesis of hyperalgesia [26,27,28] and dementia [21,22], as well as a significant overlapping in brain circuitries for neural processing of nociception and cognition [29,30,31,32], Aβ_40_ and Aβ_42_ may therefore be postulated as important biomarkers for pain intensity and cognitive dysfunction associated with knee OA. In support of this speculation, pain threshold is significantly decreased in an animal model of early AD induced by central administration of Aβ_42_ peptide [44]. Moreover, in a recent study performed with single cell RNA-seq analysis, the authors reported a strong link between the *Apolipoprotein E* (*ApoE*) gene, a major susceptibility gene associated with sporadic and familial AD [45], and chronic pain in humans by demonstrating that polymorphisms in the *ApoE* gene are associated with distinct chronic pain states [46]. We found in the present study that despite a close relationship to pain intensity, there is no apparent correlation between CSF fractalkine level and cognitive impairment in OA patients. The relationship between CSF fractalkine and cognitive dysfunction is, at best, controversial in the literature, including evidence of a range of positive [47], negative [48,49], and null [50] associations. As such, additional research is required to further clarify its role in OA-associated cognitive impairment.

There are several limitations in this study. First, a small number of OA and non-OA patients were recruited and investigated in this cross-sectional study, limiting the generalizability of the results. Nevertheless, the group sample sizes are comparable to those of previous studies that have measured pro-inflammatory mediators in CSF [24,43]. The cross-sectional design of the study constrains the capacity to ascertain causal links among OA, pain intensity, and cognitive impairment. Second, participants in the non-OA control group were not healthy subjects but patients admitted to the hospital for general surgery or urological surgery. These participants may endure concurrent systemic inflammatory reactions that could influence the comparison of pro-inflammatory molecule levels between the two groups. This might explain why the cytokine/chemokine levels in plasma, albeit lower in the non-OA group, did not reach statistical significance. We therefore include CSF measurements in the study, as it is a relevant body fluid to investigate pathophysiological processes in the CNS because of its direct contact with the brain and spinal cord. Third, the participants with OA were not matched with those without by body mass index and gender, which could potentially confound some of the biochemical measurements. In this regard, we did not find apparent differences in VAS or total CASI score, nor in the concentrations of the measured parameters between male and female participants in both OA and non-OA groups. In addition, the female-to-male ratio in the OA group is in line with its OA epidemiology [51]. Fourth, analyzing inflammatory mediators like cytokines (e.g., IL-1β, IL-6, TNF-α), chemokines (e.g., fractalkine), and AD biomarkers (e.g., Aβ_40_, Aβ_42_) in the synovial fluid from the affected joints in OA patients would reflect the local environment within the OA joint. This could complement the systemic profiles measured in CSF and plasma and yield further insights into the association between biomarkers in the local milieu of the OA joint, plasma, and CSF in the manifestation of cognitive dysfunction associated with chronic knee pain. Accordingly, this issue warrants further investigation. To this end, peripheral knee joint BDNF/TrkB signaling has been demonstrated to be engaged in the maintenance of chronic OA joint pain [52]. Lastly, it is also important to recognize that our observations were made based on VAS and CASI questionnaires for subjective assessment of pain and cognition. Although both assessments are well accepted tools, respectfully, for pain and cognition evaluation in clinical practices [53,54], VAS scales require subjects to place a quantitative rating on their pain sensation and convert a subjective feeling into a quantitative number for pain measurement, causing possible bias and lack of objectivity. The CASI is a multidimensional measure composed of nine cognitive domains that adds inter-domain complexities in detecting cognitive dysfunctions [54]. Therefore, detailed neurological (e.g., lower temporal summation of the nociceptive flexion reflex, conditioned pain modulation) and neuroimaging (e.g., brain magnetic resonance image, positron emission tomography) assessments for pain and cognition should be included in future studies to further strengthen the current findings. At the same time, further studies in a larger sample size of gender-matched participants are needed to validate the results of this study.

## 4. Materials and Methods

### 4.1. Ethics Approval and Consent

The prospective observational study was conducted between August 2019 and April 2021 at Kaohsiung Veterans General Hospital in Taiwan. This study was approved by the Institutional Review Board of Kaohsiung Veterans General Hospital (IRB No. VGHKS19-CT2-21) and registered with ClinicalTrials.gov (NCT05570240). Written informed consent was received from the OA and control patients before their participation in the study.

### 4.2. The Enrollment of Patients

A total of 50 patients (26 in the OA group and 24 in the non-OA control group) who received elective surgery requiring spinal anesthesia participated in this prospective observational study. OA was diagnosed by orthopedic physicians based on the disease diagnosis code ICD-10-CM Diagnosis Code M17, Kellgren-Lawrence grade IV [55]. The inclusion criteria of the OA group were age ≥ 20 years and overall health score of classes I–III classified by the American Society of Anesthesiologists (ASA) [56]. The exclusion criteria of OA group were age < 20 years, autoimmune diseases, previous knee injury or infection history, brain region disease (such as stroke or brain tumor), mild cognitive impairment, dementia or other neurodegenerative diseases, cancer, and with other chronic pain.

Patients in the non-OA control group were included based on ASA score of class I–III, age ≥ 20 years, and scheduled to receive elective general surgery or urological surgery with spinal anesthesia such as hernioplasty, ureteroscopy and laser stone fragmentation, and transurethral resection of the prostate. These patients did not show signs of infection, autoimmune diseases, or allergies. The exclusion criteria of control group are the same as those of the OA group patients plus with OA pain. Throughout the study, prescription drugs used by the patients were not adjusted.

### 4.3. Pain and Cognitive Function Evaluations

All patients were asked to evaluate their current pain intensity by experienced nurses using the visual analogue scale (VAS, ranged from 0–10, no moving or standing) [57] one day before surgery. On the same day, all patients also received the Cognitive Abilities Screening Instrument (CASI) [54] questionnaires. The VAS and CASI are well accepted questionnaires respectfully for pain and cognition evaluation in clinical practices. The questionnaire used was the Chinese version of CASI (CASI C-2.0) [58], which has been commonly used in many clinical and epidemiological studies in Taiwan for dementia research and in clinical practice to evaluate a subject’s cognitive abilities.

The CASI II includes nine cognitive domains: attention, mental manipulation, short- term memory, long-term memory, orientation, language, drawing, abstract thinking and judgment, and animal-name fluency. The total score of the CASI was adjusted to one of four percentiles (i.e., P5, P10, P20, and P50) according to different education ranges and ages. The higher percentile score indicates better cognitive performance. In clinical practice, the cutoff percentile scores less than P5 are diagnosed as cognitive functions below the normal limit of the same age and education level [58].

### 4.4. Blood and CSF Sample Collections

All participants enrolled in this study received spinal anesthesia. Blood samples of 10 mL were obtained before anesthesia. CSF of 3 mL was obtained by lumbar puncture prior to the administration of local analgesics into the spinal dura sac. All participants were evaluated for post-lumbar puncture-related complications such as back pain or headache. No patient complained of complications.

### 4.5. Parameter Measurements of Plasma and the CSF Samples

The levels of monocyte chemoattractant protein-1 (MCP-1) and transforming growth factor-β (TGF-β) in plasma and CSF were measured by multiplex immunoassay performed by the Inflammation Core Facility (Institute of Biomedical Sciences, Academia Sinica, Taipei, Taiwan, supported by AS-CFII-111-213). Antibody-conjugated magnetic beads were incubated with cytokine-containing samples, washed, and incubated with biotinylated antibody and subsequently with streptavidin-phycoerythrin. The fluorescence levels of the beads were measured by the Bio-Plex^®^ 200 system (Bio-Rad, Hercules, CA, USA), and the concentration of the chemokines were calculated with standard solutions. All assays were protected from light and performed at room temperature.

The plasma and CSF levels of interleukin-1β (IL-1β), IL-6, tumor necrosis factor-α (TNF-α), brain-derived neurotrophic factor (BDNF), and fractalkine, as well as CSF levels of Aβ_40_, Aβ_42_, total tau (t-tau), and phosphorylated tau at threonine^181^ (p-tau), were assayed using the commercially available MILLIPLEX^®^ MAP panel kit (Millipore, Billerica, MA, USA), according to the manufacturer’s instructions.

### 4.6. Statistical Analysis

Group differences on background variables, including demographic data, VAS pain scores, and CASI cutoff percentile, of OA and non-OA patients were compared using the unpaired Student’s *t*-test or the chi-squared test. If a variable was found to be different, this was considered as a potential control variable in the following analyses. Comparisons of plasma and CSF levels of biochemical parameters between the two groups were performed using the unpaired Student’s *t*-test and adjusted for covariates with multivariate analysis of covariance (MANCOVA) models [59]. Control variables were entered as covariates in these models. Correlations between plasma or CSF substances levels and VAS or CASI score were assessed using Pearson’s correlation coefficient [60], which provides a measure of the strength of the linear association between two variables, and simple linear regression analysis [61], which measures the linear relationship between a predictor and an outcome variable. Results are presented as correlation coefficients (R) with 95% confidence intervals (CIs) and p values. All data are presented as means ± SD. The criterion for statistical significance was *p* < 0.05. All the statistical analyses and figures were performed in SPSS 22 (IBM, New York, NY, USA) and GraphPad Prism software version 9 (GraphPad Software Inc., La Jolla, CA, USA).

## 5. Conclusions

In conclusion, this study was performed to explore the associations among pro-inflammatory molecules, AD biomarkers, pain intensity, and cognitive function in patients with knee OA. Our observations suggest that poor cognitive performance is associated with knee OA, and this association is particularly pronounced in OA patients with chronic pain. Our data also imply that CSF AD biomarkers, such as Aβ_40_ and Aβ_42_, may partially mediate this relationship. The current observations may pave the way for future research to better understand the cognitive domains and their relationship with pain to improve therapeutic management and avoid unfavorable cognitive outcomes in OA. Furthermore, in the perspective of developing novel therapeutic strategies relevant to precision medicine in treatment for cognitive impairment associated with OA, patient Aβ biomarkers evaluation may represent an advantageous approach.

## Figures and Tables

**Table 1:** Demographic characteristics and comorbidities of non-OA control and OA participants.

	Control Group	OA Group		*p* Value
No.	24	26		
Age, years, mean (SD)	63.00 (±11.865)	68.58 (±7.70)	T = −1.987 ^a^	0.053
Gender, *n* (%)			X^2^ = 19.284 ^b^	<0.001 *
Female	**2 (8.4)**	**18 (69.2)**		
Male	22 (91.6)	8 (30.8)		
Marital status, *n* (%)			X^2^ = 2.279 ^b^	0.182
Never married	4 (16.7)	1 (3.8)		
Married	20 (83.3)	25 (96.2)		
Education years, mean (SD)	**13.63 (±2.78)**	**8.42 (±4.04)**	t = 5.260 ^a^	**<0.001 ^#^**
Body weight, kg, mean (SD)	70.8 (±9.09)	68.87 (±15.22)	t = 0.539 ^a^	0.592
BMI, kg/m^2^, mean (SD)	**24.99 (±2.83)**	**28.43 (±4.61)**	t = −3.202 ^a^	**0.003 ^#^**
Smoking, *n* (%)	1 (4.2)	0 (0)	X^2^ = 1.105 ^b^	0.480
Drinking, *n* (%)	2 (8.3)	5 (19.2)	X^2^ = 1.231 ^b^	0.420
Comorbidities, *n* (%)				
Hypertension	9 (37.5)	12 (46.2)	X^2^ = 0.384 ^b^	0.578
Diabetes mellitus	4 (16.7)	8 (30.8)	X^2^ = 1.361 ^b^	0.327
Dyslipidemia	1 (4.2)	3 (11.5)	X^2^ = 0.921 ^b^	0.611
Coronary artery disease	1(4.2)	2 (7.7)	X^2^ = 0.275 ^b^	>0.99

Data are means ± standard deviation (SD). ^#,a^ Statistically significant vs. control group by Student’s *t*-test; *^,b^ statistically significant vs. control group by chi-squared test. BMI, body mass index; OA, osteoarthritis. **Bold**, statistically significant.

**Table 2:** VAS pain score and CASI cutoff percentile of non-OA control and OA participants.

	Control Group	OA Group		*p* Value
No.	24	26		
VAS, mean (SD)	**0.21 (±0.509)**	**2.38 (±0.852)**	t = −11.059 ^a^	**<0.001 ^#^**
CASI cutoff percentile, *n* (%)				
<P5	**1 (4.20)**	**7 (26.9)**	X^2^ = 4.809 ^b^	0.028 *
P5	**0 (0)**	**4 (15.4)**	X^2^ = 4.013 ^b^	0.045 *
P10	0 (0)	2 (7.70)	X^2^ = 1.923 ^b^	0.166
P20	6 (25.0)	3 (11.5)	X^2^ = 1.532 ^b^	0.216
P50	17 (70.80)	10 (38.5)	X^2^ = 5.265 ^b^	0.022 *

Data are means ± standard deviation (SD). ^#,a^ Statistically significant vs. non-OA control group by Student’s *t*-test; *^,b^ statistically significant vs. non-OA control group by chi-squared test. CASI, Cognitive Abilities Screening Instrument; OA, osteoarthritis; VAS, visual analogue scale. Please note that the total score of the CASI is adjusted as one of four percentiles (P5, P10, P20, P50) according to different education ranges and ages, and the cutoff percentile scores less than P5 are regarded as below the normal limit of the same age and education level. **Bold**, statistically significant.

**Table 3:** Plasma concentrations of cytokines/chemokines in non-OA control and OA participants.

	Control Group (n = 24)	OA Group (n = 26)	Student’s *t*-Test *p* Value	MANCOVA	
				F	*p* Value
Plasma, pg/mL, mean (SD)					
IL-1β	9.18 (±10.62)	12.17 (±18.02)	0.483	0.827	0.515
IL-6	1.90 (±5.29)	2.88 (±6.98)	0.583	1.173	0.336
TNF-α	15.07 (±16.36)	17.19 (±11.46)	0.596	0.778	0.545
BDNF	489.32 (±838.06)	554.08 (±897.81)	0.794	0.132	0.970
Fractalkine	73.52 (±51.67)	79.98 (±40.81)	0.624	0.237	0.916
MCP-1	70.02 (±31.22)	82.74 (±77.34)	0.456	0.720	0.583
TGF-β	4627.29 (±4185.06)	7322.43 (±8088.27)	0.143	1.443	0.235

Data are means ± standard deviation (SD). There is no significant difference among groups by Student’s *t*-test or by a multivariate analysis of covariance (MANCOVA) adjusted for covariates (gender, education years, and body mass index). BDNF, brain-derived neurotrophic factor; IL-1β, interleukin-1β; IL-6, interleukin-6; MCP-1, monocyte chemoattractant protein-1; OA, osteoarthritis; TGF-β, transforming growth factor beta, TNF-α, tumor necrosis factor alpha.

**Table 4:** Concentrations of cytokine/chemokine and AD biomarkers in cerebrospinal fluid of control and OA participants.

	Control Group (n = 24)	OA Group (n = 26)	Student’s *t*-Test *p* Value	MANCOVA	
				F	*p* Value
CSF, pg/mL, mean (SD)					
IL-1β	1.42 (±0.83)	1.10 (±0.46)	0.093	1.129	0.355
IL-6	1.86 (±0.79)	2.55 (±1.42)	0.041 *	2.376	0.066
TNF-α	**1.32 (±0.34)**	**1.69 (±0.52)**	0.004 *	3.106	**0.024 ^#^**
BDNF	**0.42 (±0.11)**	**0.71 (±0.44)**	0.003 *	3.045	**0.026 ^#^**
Fractalkine	**36.19 (±10.80)**	**52.72 (±20.69)**	0.001 *	3.424	**0.016 ^#^**
MCP-1	466.98 (±121.21)	474.17 (±92.09)	0.813	0.257	0.904
TGF-β	65.83 (±15.33)	81.09 (±23.60)	0.009 *	2.407	0.063
Aβ_40_	**1953.21 (±492.02)**	**2390.17 (±876.10)**	0.034 *	3.579	**0.013 ^#^**
Aβ_42_	**315.26 (±79.75)**	**395.37 (±135.48)**	0.014 *	4.875	**0.002 ^#^**
t-tau	199.32 (±73.79)	195.68 (±67.01)	0.856	0.415	0.797
p-tau	50.39 (±16.88)	55.15 (±19.42)	0.361	0.438	0.780

Data are means ± standard deviation (SD). * Statistically significant vs. non-OA control group by Student’s *t*-test, adjusted by a multivariate analysis of covariance (MANCOVA) for covariates (gender, education years, and body mass index); ^#^ statistically significant vs. non-OA control group by MANCOVA. Aβ_40_, amyloid-β_40_; Aβ_42_, amyloid-β_42_; BDNF, brain-derived neurotrophic factor; IL-1β, Interleukin-1β; IL-6, interleukin-6; MCP-1, monocyte chemoattractant protein-1; OA, osteoarthritis; t-tau, total-tau; p-tau, phosphorylated tau at theronine^181^; TGF-β, transforming growth factor beta; TNF-α, tumor necrosis factor alpha. **Bold**, statistically significant.

**Table 5:** Correlation of VAS pain score with CASI cognition score in OA participants.

Cognitive Domains of CASI	VAS Score	
	Pearson Correlation (R) [95% CI]	*p* Value (2-Tailed)
Total score	**−0.514 [−0.752, −0.158]**	0.007 *
LTM	−0.177 [−0.528, 0.226]	0.388
STM	**−0.426 [−0.698, −0.046]**	0.030 *
ATTEN	**−0.394 [−0.678, −0.008]**	0.047 *
MENMA	**−0.626 [−0.816, −0.315]**	0.001 *
ORIEN	−0.373 [−0.664, 0.017]	0.060
ABSTR	**−0.510 [−0.749, −0.153]**	0.008 *
LANG	−0.312 [−0.624, 0.086]	0.121
DRAW	−0.146 [−0.505, 0.256]	0.477
ANML	−0.358 [−0.655, 0.034]	0.073

* Statistically significant between VAS and CASI scores by Pearson correlation coefficient and simple linear regression analysis (n = 26). Correlation coefficients are followed by 95% CI. ABSTR, abstract thinking and judgment; ANML, animal-name fluency; ATTEN, attention; CASI, Cognitive Abilities Screening Instrument; CI, confidence interval; DRAW, drawing; LANG, language; LTM, long-term memory; MENMA, mental manipulation; OA, osteoarthritis; ORIEN, orientation; STM, short-term memory; VAS, visual analogue scale. **Bold**, statistically significant.

**Table 6:** Correlation between blood and CSF biochemical parameters with VAS pain score in OA participants.

Plasma Molecule	VAS Score		CSF Molecule	VAS Score	
	Pearson Correlation (R) [95% CI]	*p* Value (2-Tailed)		Pearson Correlation (R) [95% CI]	*p* Value (2-Tailed)
IL-1β	−0.252 [−0.583, 0.150]	0.215	IL-1β	0.018 [−0.372, 0.403]	0.929
IL-6	−0.295 [−0.612, 0.104]	0.144	IL-6	**−0.439 [−0.706, −0.062]**	0.025 *
TNF-α	−0.257 [−0.586, 0.145]	0.206	TNF-α	0.155 [−0.247, 0.512]	0.450
BDNF	0.137 [−0.264, 0.498]	0.505	BDNF	0.025 [−0.366, 0.408]	0.903
Fractalkine	−0.141 [−0.501, 0.261]	0.491	Fractalkine	**0.423 [0.043, 0.696]**	0.031 *
MCP-1	0.224 [−0.179, 0.563]	0.271	MCP-1	0.141 [−0.261, 0.501]	0.492
TGF-β	0.306 [−0.092, 0.620]	0.129	TGF-β	0.383 [−0.005, 0.671]	0.053
			Aβ_40_	**0.631 [0.323, 0.818]**	0.001 *
			Aβ_42_	**0.610 [0.292, 0.807]**	0.001 *
			t-tau	0.114 [−0.286, 0.480]	0.579
			p-tau	0.273 [−0.128, 0.597]	0.178

* Statistically significant by Pearson correlation coefficient and simple linear regression analysis (n = 26). Correlation coefficients are followed by 95% CI. Aβ_40_, amyloid-β_40_; Aβ_42_, amyloid-β_42_; BDNF, brain-derived neurotrophic factor; CI, confidence interval; CSF, cerebrospinal fluid; IL-1β = Interleukin-1β; IL-6 = interleukin-6; MCP-1, monocyte chemoattractant protein-1; OA, osteoarthritis; t-tau, total-tau; p-tau, phosphorylated tau at theronine^181^; TGF-β, transforming growth factor beta; TNF-α, tumor necrosis factor alpha. **Bold**, statistically significant.

**Table 7:** Correlation between CSF Aβ40 and Aβ42 with CASI cognition score in OA participants.

Cognitive Domains of CASI	CSF Aβ_40_		Cognitive Domains of CASI	CSF Aβ_42_	
	Pearson Correlation (R) [95% CI]	*p* Value (2-Tailed)		Pearson Correlation (R) [95% CI]	*p* Value (2-Tailed)
Total score	−0.357 [−0.654, 0.035]	0.074	Total score	−0.365 [−0.659, 0.026]	0.067
LTM	−0.313 [−0.625, 0.085]	0.120	LTM	−0.356 [−0.653, 0.036]	0.074
STM	−0.188 [−0.536, 0.215]	0.358	STM	−0.176 [−0.527, 0.227]	0.389
ATTEN	**−0.464 [−0.722, −0.093]**	0.017 *	ATTEN	**−0.441 [−0.708, −0.065]**	0.024 *
MENMA	−0.344 [−0.645, 0.050]	0.085	MENMA	−0.370 [−0.662, 0.020]	0.063
ORIEN	−0.258 [−0.587, 0.144]	0.203	ORIEN	−0.291 [−0.610, 0.109]	0.149
ABSTR	**−0.446 [−0.711, −0.071]**	0.022 *	ABSTR	**−0.459 [−0.719, −0.087]**	0.018 *
LANG	−0.177 [−0.528, 0.226]	0.387	LANG	−0.204 [−0.548, 0.199]	0.317
DRAW	−0.038 [−0.419, 0.355]	0.855	DRAW	−0.051 [−0.430, 0.343]	0.804
ANML	−0.244 [−0.577, 0.158]	0.230	ANML	−0.195 [−0.542, 0.208]	0.339

* Statistically significant by Pearson correlation coefficient and simple linear regression analysis (n = 26). Correlation coefficients are followed by 95% CI. ABSTR, abstract thinking and judgment; Aβ_40_, amyloid-β_40_; Aβ_42_, amyloid-β_42_; ANML, animal-name fluency; ATTEN, attention; CASI, Cognitive Abilities Screening Instrument; CI, confidence interval; CSF, cerebrospinal fluid; DRAW, drawing; LANG, language; LTM, long-term memory; MENMA, mental manipulation; OA, osteoarthritis; ORIEN, orientation; STM, short-term memory. **Bold**, statistically significant.

## Data Availability

The data that support the findings of this study are available from the corresponding author.

## Supplementary Materials

The following supporting information can be downloaded at https://www.mdpi.com/article/10.3390/ijms252312575/s1.
