# Supplementary material for: Brain Amyloid-β Peptide Is Associated with Pain Intensity and Cognitive Dysfunction in Osteoarthritic Patients
Source: Int J Mol Sci. 2024 Nov 22;25(23):12575. doi: 10.3390/ijms252312575 (PMC11641244; doi:10.3390/ijms252312575)
Supplement: Supplementary file 1 [file ijms-25-12575-s001.zip › ijms-3330990-supplementary.pdf]

Supplementary Materials

Brain Amyloid-β Peptide Is Associated with Pain Intensity and Cognitive Dysfunction in Osteoarthritic Patients

Chun-Hsien Wen[1,2,3,4,5*], Hong-Yo Kang[1,6,7], Julie Y.H. Chan[8*]

[1] Graduate Institute of Clinical Medical Sciences, Chang Gung University, Taoyuan, 333, Taiwan.; hkang3@gap.cgu.edu.tw (H.Y.K.)

[2] Department of Anesthesiology, Kaohsiung Veterans General Hospital, Kaohsiung, 813414, Taiwan.

[3] Department of Nursing, Shu-Zen Junior College of Medicine and Management, Kaohsiung, 82144, Taiwan

[4] Department of Nursing, Meiho University, Pingtung, 912009, Taiwan.

[5] School of Medicine, National Yang Ming Chiao Tung University, Taipei, 112304, Taiwan.

[6] Division of Endocrinology and Metabolism, Department of Internal Medicine, Kaohsiung Chang Gung Memorial Hospital, Kaohsiung, 833401, Taiwan

[7] College of Medicine, Chang Gung University, Taoyuan, 333, Taiwan.

[8] Institute for Translational Research in Biomedicine, Kaohsiung Chang Gung Memorial Hospital, Kaohsiung, 833401, Taiwan.

Table S1.

Plasma concentrations of cytokines/chemokines in the male and female non-OA and OA participants

	Non-OA group				OA group			
	**Male**	**Female**		***P* value**	**Male**	**Female**		***P* value**
No.	22	2			8	18		
Plasma, pg/mL, mean (SD)								
IL-1β	7.859 (±8.098)	23.725 (±27.443)	t=0.814	0.563	20.216(±29.214)	8.594(±9.058)	t=-1.102	0.304
IL-6	2.017(±5.523)	0.645(±0.488)	t=-0.344	0.734	5.599(±12.235)	1.668(±2.246)	t=-0.902	0.396
TNF-α	12.328(±7.828)	45.230(±53.712)	t=0.865	0.545	22.786(±18.277)	14.699(±5.846)	t=-1.224	0.257
BDNF	516.893(±871.565)	186.005 (±29.790)	t=-0.526	0.604	527.350(±841.815)	565.959 (±945.046)	t=0.099	0.922
Fractalkine	72.674(±53.808)	82.850(±20.294)	t=0.261	0.796	87.048(±48.502)	76.843(±38.033)	t=-0.581	0.567
MCP-1	70.209(±32.647)	67.960(±4.695)	t=-0.095	0.925	52.928(±32.423)	95.991(±88.086)	t=1.33	0.196
TGF-β	4345.668(±3974.297)	7725.115(±7085.542)	t=1.098	0.284	4265.701(±3011.362)	8680.972(±9280.138)	t =1.185	0.083

Data are mean ± standard deviation (SD). There is no significant difference among groups by Student's *t* test; BDNF, brain-derived neurotrophic factor; IL-1β, interleukin-1β; IL-6, interleukin-6; MCP-1, monocyte chemoattractant protein-1; OA, osteoarthritis; TGF-β, transforming growth fac-tor Beta, TNF-α, tumor necrosis factor alpha.

Table S2

Concentrations of cytokine/chemokine and AD biomarkers in cerebrospinal fluid in the male and female non-OA and OA participants

	Non-OA group				OA group			
	**Male**	**Female**		***P* value**	**Male**	**Female**		***P* value**
No.	22	2			8	18		
CSF, pg/mL, mean (SD)								
IL-1β	1.414(±0.866)	1.445(±0.346)	t=0.049	0.962	1.004(±0.523)	1.136(±0.444)	t=0.663	0.514
IL-6	1.906(±0.805)	1.295(±0.106)	t=-1.053	0.304	3.238(±1.655)	2.239(±1.227)	t=-1.721	0.098
TNF-α	1.286(±0.332)	1.670(±0.071)	t=1.604	0.123	1.739(±0.474)	1.673(±0.556)	t=-0.291	0.774
BDNF	0.412(±0.114)	0.450(±0.057)	t=0.456	0.653	0.720(±0.501)	0.710(±0.433)	t=-0.052	0.959
Fractalkine	35.162(±10.038)	47.545(±16.907)	t=1.605	0.123	49.784(±21.629)	54.023(±20.765)	t=0.475	0.639
MCP-1	467.558(±120.526)	460.595(±181.069)	t=--0.076	0.940	441.008(±114.658)	488.905(±79.446)	t=1.237	0.228
TGF-β	67.286(±15.188)	49.805(±0.530)	t=-1.595	0.125	77.734(±23.259)	82.587(±24.259)	t=0.476	0.638
$A\beta_{40}$	1958.546(±487.621)	1894.500(±753.069)	t=-0.172	0.865	1944.314(±564.690)	2588.333(±928.611)	t=1.807	0.083
$A\beta_{42}$	310.645(±72.654)	366.040(±172.605)	t=0.938	0.358	336.328(±112.789)	421.608(±139.234)	t=1.520	0.142
t-tau	205.092(±69.116)	135.805(±126.890)	t=-1.290	0.211	188.194(±52.156)	199.002(±73.794)	t=0.373	0.712
p-tau	51.296(±16.324)	40.380(±27.139)	t=-0.871	0.393	46.623(±9.160)	58.941(±21.688)	t=1.533	0.138

Data are mean ± standard deviation (SD). There is no significant difference among groups by Student's *t* test; $A\beta_{40}$, amyloid-β_{40}; $A\beta_{42}$, amyloid-β_{42}; BDNF, brain-derived neurotrophic factor; CSF, cerebrospinal fluid; IL-1β, In-terleukin-1β; IL-6, interleukin-6; MCP-1, monocyte chemoattractant protein-1; OA, osteoarthritis; t-tau, total-tau; p-tau, phosphorylated tau at theronine^{181}; TGF-β, transforming growth factor beta; TNF-α, tumor necrosis factor alpha.

Table S3

Correlation between VAS pain score and CASI cognition score in non-OA participants

Cognitive domains of CASI	VAS score	
	Pearson Correlation (R) [95% CI]	*p* value (2-tailed)
Total score	-0.187 [-0.549, 0.234]	0.382
LTM	-0.030[-0.428, 0.378]	0.891
STM	0.043 [-0.367, 0.438]	0.843
ATTEN	0.007 [-0.398, 0.049]	0.974
MENMA	-0.278 [-0.612, 0.142]	0.189
ORIEN	-0.281 [-0.615, 0.138]	0.183
ABSTR	-0.230 [-0.580, 0.191]	0.280
LANG	-0.030 [-0.428, 0.378]	0.891
DRAW	-0.135 [-0.511, 0.284]	0.529
ANML	-0.051 [-0.445, 0.360]	0.813

No significant difference between CASI and VAS scores by Pearson correlation coefficient and simple linear regression analysis (n=24). Correlation coefficients are followed by 95% CI. ABSTR, abstract thinking and judgment; $A\beta_{40}$, amyloid-β_{40}; $A\beta_{42}$, amyloid-β_{42}; ANML, animal-name fluency; ATTEN, attention; CASI, Cognitive Abilities Screening Instrument; CI, confidence interval; CSF, cerebrospinal fluid; DRAW, drawing; LANG, language; LTM, long-term memory; MENMA, mental manipulation; OA, osteoarthritis; ORIEN, orientation; STM, short-term memory; VAS, Visual Analogue Scale.

Table S4

Correlation between blood and CSF parameter with VAS pain score in non-OA participants

	VAS score			VAS score	
**Plasma molecule**	Pearson Correlation (R) [95% CI]	*p* value (2-tailed)	**CSF molecule**	Pearson Correlation (R) [95% CI]	*p* value (2-tailed)
IL-1β	-0.285 [-0.617, 0.134]	0.177	IL-1β	-0.007 [-0.409, 0.398]	0.975
IL-6	-0.091 [-0.477, 0.324]	0.674	IL-6	-0.105 [-0.488, 0.312]	0.626
TNF-α	-0.203 [-0.561, 0.218]	0.341	TNF-α	0.222 [-0.199, 0.574]	0.297
BDNF	0.312 [-0.105, 0.635]	0.138	BDNF	0.235 [-0.186, 0.583]	0.268
Fractalkine	-0.149 [-0.521, 0.271]	0.488	Fractalkine	0.000 [-0.403, 0.403]	0.999
MCP-1	-0.203 [-0.561, 0.218]	0.342	MCP-1	-0.245 [-0.590, 0.176]	0.249
TGF-β	0.059 [-0.353, 0.452]	0.784	TGF-β	0.439 [0.044, 0.716]	0.032*
			$A\beta_{40}$	0.282 [-0.137, 0.615]	0.182
			$A\beta_{42}$	0.123 [-0.295, 0.502]	0.567
			t-tau	0.344 [-0.069, 0.656]	0.100
			p-tau	0.124 [-0.294, 0.502]	0.565

*Statistically significant between plasma or CSF biomarkers with VAS pain score by Pearson correlation coefficient and simple linear regression analysis (n=24). Correlation coefficients are followed by 95% CI. $A\beta_{40}$, amyloid-β_{40}; $A\beta_{42}$, amyloid-β_{42}; BDNF, brain-derived neurotrophic factor; CI, confidence interval; CSF, cerebrospinal fluid; IL-1β, interleukin-1β; IL-6, interleukin-6; MCP-1, monocyte chemoattractant protein-1; OA, osteoarthritis; t-tau, total-tau; p-tau, phosphorylated tau at theronine^{181}; TGF-β, transforming growth factor beta; TNF-α, tumor necrosis factor alpha, VAS, Visual Analogue Scale.

Table S5

Correlation between CSF TNF-α and fractalkine with CASI cognition score in OA participants

Cognitive domains of CASI	CSF TNF-α		Cognitive domains of CASI	CSF fractalkine	
	Pearson Correlation (R) [95% CI]	*p* value (2-tailed)		Pearson Correlation (R) [95% CI]	*p* value (2-tailed)
Total score	-0.122 [-0.486, 0.279]	0.554	Total score	-0.149 [-0.507, 0.253]	0.468
LTM	0.054 [-0.341, 0.432]	0.795	LTM	0.038 [-0.355, 0.419]	0.854
STM	-0.139 [-0.500, 0.263]	0.499	STM	-0.125 [-0.355, 0.419]	0.544
ATTEN	0.151 [-0.251, 0.509]	0.463	ATTEN	0.069 [-0.327, 0.445]	0.738
MENMA	-0.298 [-0.614, 0.101]	0.139	MENMA	-0.338 [-0.641, 0.057]	0.092
ORIEN	-0.017 [-0.402, 0.373]	0.935	ORIEN	-0.121 [-0.486, 0.279]	0.557
ABSTR	-0.260 [-0.588, 0.142]	0.199	ABSTR	-0.288 [-0.608, 0.112]	0.154
LANG	0.036 [-0.356, 0.418]	0.860	LANG	0.029 [-0.362, 0.412]	0.890
DRAW	-0.054 [-0.432, 0.341]	0.794	DRAW	-0.046 [-0.426, 0.348]	0.824
ANML	-0.109 [-0.476, 0.291]	0.595	ANML	-0.070 [-0.445, 0.326]	0.734

No significant difference between TNF-α or fractalkine and CASI scores by Pearson correlation coefficient and simple linear regression analysis (n=26). Correlation coefficients are followed by 95% CI. ABSTR, abstract thinking and judgment; ANML, animal-name fluency; ATTEN, attention; CASI, Cognitive Abilities Screening Instrument; CI, confidence interval; CSF, cerebrospinal fluid; DRAW, drawing; LANG, language; LTM, long-term memory; MENMA, mental manipulation; OA, osteoarthritis; ORIEN, orientation; STM, short-term memory; TNF-α, tumor necrosis factor alpha.

Table S6

Correlation between CSF TNF-α and fractalkine with CASI cognition score in non-OA participants

Cognitive domains of CASI	CSF TNF-α		Cognitive domains of CASI	CSF fractalkine	
	Pearson Correlation (R) [95% CI]	*p* value (2-tailed)		Pearson Correlation (R) [95% CI]	*p* value (2-tailed)
Total score	0.222 [-0.199, 0.574]	0.296	Total score	-0.123[-0.502, 0.295]	0.566
LTM	0.019[-0.388, 0.419]	0.930	LTM	-0.114[-0.495, 0.303]	0.595
STM	0.308 [-0.109, 0.633]	0.143	STM	-0.101 [-0.484, 0.316]	0.641
ATTEN	-0.151 [-0.522, 0.269]	0.482	ATTEN	0.242 [-0.588, 0.179]	0.255
MENMA	0.287 [-0.132, 0.619]	0.175	MENMA	0.126 [-0.293, 0.504]	0.558
ORIEN	0.130 [-0.289, 0.507]	0.546	ORIEN	0.023 [-0.384, 0.421]	0.915
ABSTR	-0.119 [-0.499, 0.299]	0.581	ABSTR	0.031 [-0.377, 0.429]	0.884
LANG	-0.178 [-0.542, 0.243]	0.407	LANG	-0.044 [-0.439, 0.366]	0.839
DRAW	0.251 [-0.170, 0.594]	0.237	DRAW	0.016 [-0.417, 0.390]	0.942
ANML	-0.047 [-0.442, 0.363]	0.829	ANML	-0.347 [-0.658, 0.065]	0.097

No significant difference between TNF-α or fractalkine and CASI scores by Pearson correlation coefficient and simple linear regression analysis (n=24). Correlation coefficients are followed by 95% CI. ABSTR, abstract thinking and judgment; ANML, animal-name fluency; ATTEN, attention; CASI, Cognitive Abilities Screening Instrument; CI, confidence interval; CSF, cerebrospinal fluid; DRAW, drawing; LANG, language; LTM, long-term memory; MENMA, mental manipulation; OA, osteoarthritis; ORIEN, orientation; STM, short-term memory; TNF-α, tumor necrosis factor alpha.

Table S7

Correlation between CSF Aβ40 and Aβ42 with CASI cognition score in non-OA participants

Cognitive domains of CASI	CSF $A\beta_{40}$		Cognitive domains of CASI	CSF $A\beta_{42}$	
	Pearson Correlation (R) [95% CI]	*p* value (2-tailed)		Pearson Correlation (R) [95% CI]	*p* value (2-tailed)
Total score	-0.264 [-0.603, 0.156]	0.213	Total score	-0.095 [-0.480, 0.321]	0.660
LTM	0.087 [-0.328, 0.474]	0.686	LTM	0.218 [-0.203, 0.571]	0.306
STM	-0.009 [-0.411, 0.396]	0.965	STM	0.089 [-0.326, 0.475]	0.679
ATTEN	-0.217 [-0.571, 0.204]	0.307	ATTEN	-0.231 [-0.580, 0.191]	0.278
MENMA	-0.253 [-0.596, 0.167]	0.232	MENMA	-0.121 [-0.500, 0.297]	0.574
ORIEN	-0.148 [-0.520, 0.272]	0.491	ORIEN	0.110 [-0.307, 0.492]	0.609
ABSTR	-0.079 [-0.468, 0.335]	0.712	ABSTR	-0.057 [-0.450, 0.355]	0.792
LANG	-0.191 [-0.552, 0.230]	0.371	LANG	-0.316 [-0.638, 0.100]	0.133
DRAW	-0.288 [-0.620, 0.130]	0.172	DRAW	-0.130 [-0.507, 0.288]	0.544
ANML	-0.204 [-0.561, 0.218]	0.340	ANML	-0.235 [-0.583, 0.186]	0.268

No significant difference between $A\beta_{40}$ or $A\beta_{42}$ and CASI scores by Pearson correlation coefficient and simple linear regression analysis (n=24). Correlation coefficients are followed by 95% CI. ABSTR, abstract thinking and judgment; $A\beta_{40}$, amyloid-β_{40}; $A\beta_{42}$, amyloid-β_{42}; ANML, animal-name fluency; ATTEN, attention; CASI, Cognitive Abilities Screening Instrument; CI, confidence interval; CSF, cerebrospinal fluid; DRAW, drawing; LANG, language; LTM, long-term memory; MENMA, mental manipulation; OA, osteoarthritis; ORIEN, orientation; STM, short-term memory.
